# Towards the development of actionable recommendations for improving mental healthcare access for migrants: a qualitative study in Munich, Germany

**DOI:** 10.1186/s12889-026-26673-1

**Published:** 2026-02-25

**Authors:** Sophia Baierl, Zeliha Öcek, Caroline Jung-Sievers, Michaela Coenen

**Affiliations:** 1 Chair for Public Health and Health Services Research, Institute of Medical Information Processing, Biometry and Epidemiology (IBE), Faculty of Medicine, LMU Munich, Munich, Germany; 2 Pettenkofer School of Public Health, Munich, Germany

**Keywords:** Migrants, Mental health, Access to healthcare, Barriers, Recommendations for action, Qualitative study, Determinants, Integrated care

## Abstract

**Background:**

The experience of migration is often associated with stressors affecting mental health. Furthermore, migrants face barriers to accessing mental healthcare. This study aims to explore the determinants influencing mental healthcare access for migrants in Munich, Germany, and to serve as a starting point for developing recommendations for action.

**Methods:**

The qualitative study examined the perspectives of migrants and health professionals. Former were derived from a secondary analysis of interview data collected in the SonarGlobal project in 2021. Semi-structured interviews with health professionals were conducted in 2023. Data from both groups were analysed iteratively using a shared coding structure for content analysis. Levesque et al.’s framework guided the analysis of healthcare access and was adapted by schematizing the results. The adapted framework served as the basis for developing action-oriented recommendations based on migrants’ suggestions, refined and completed by health professionals’ data. Recommendations for improving migrants’ access to mental healthcare were organised thematically and then assigned to the levels of integrated care according to Valentijn et al.: micro, meso and macro.

**Results:**

The study included 24 migrants of SonarGlobal and seven health professionals. The dimensions of mental healthcare access encompassed: (1) perceiving mental health problems; (2) ability to seek mental healthcare, including knowledge about the new healthcare system and social support; (3) acceptability of mental health services, involving provider identity and gender; (4) availability and affordability of mental health services, including insurance coverage, bureaucratic processes, and capacity and geographical distribution of services; (5) appropriateness of mental health services with providers’ and patients’ understanding of mental healthcare, and providers’ competence and capacity. Language exhibited a strong interplay across all dimensions. The analysis yielded 17 recommendations. Macro-level recommendations address discrimination and inequality. Meso-level recommendations included enhancing care capacity and coordination, training healthcare workers, and eliminating language barriers. Micro-level recommendations included activities to promote mental health.

**Conclusions:**

The determinants of migrants’ mental healthcare access are shaped by intersecting vulnerability mechanisms and systemic barriers. Improving access requires actions on micro, meso and macro level of care. This study’s recommendations offer a starting point for developing comprehensive, evidence-based strategies to ensure equitable mental healthcare access for all.

**Supplementary Information:**

The online version contains supplementary material available at 10.1186/s12889-026-26673-1.

## Background

Migration is a highly complex experience shaped by diverse motives and consequences for each person [1]. Its process involves structures and situations that can create significant challenges for migrants’ health and well-being [2]. Discrimination, poverty, structural adjustment programs, war and conflicts that force people to leave their homes, as well as exposure to violence and human rights abuses on the migration route, contribute to many migrants already facing mental health problems when they arrive in the host countries [1–4]. Upon arrival, migrants may experience situational vulnerability resulting from xenophobia, social exclusion, precarious living and working conditions, legal uncertainty, and restricted access to fundamental rights, including healthcare, all of which stem from structural inequalities and systemic racism [2, 3, 5–8]. The intersectionality of these vulnerability mechanisms, along with language difficulties and adapting to a new environment, can exacerbate stress and vulnerability to mental health problems [5, 9, 10].

A substantial part of migrants experience symptoms such as anxiety, sadness, hopelessness, stress, sleep disturbances or anger as a result of migration [3, 11]. Studies indicate a higher prevalence of common mental disorders, such as depression and post-traumatic stress disorder (PTSD) among migrant populations compared to the general population of the host communities [3, 5, 11, 12]. Certain migrant groups are also more prone to psychotic disorders than the resident population [3, 11, 12]. Despite this heightened mental health burden, many migrants face significant barriers in accessing healthcare services, particularly mental healthcare. These barriers include a lack of awareness of available services, communication challenges, stigma, legal restrictions, and the absence of migrant-sensitive healthcare options [13–16]. A critical social resilience perspective, as advocated by Preston et al., underscores that mental health problems and barriers to healthcare access stem from institutional inequalities rather than a lack of individual capacity [17]. Therefore, improving the health of newcomers requires strengthening community and institutional support while addressing the structural barriers that hinder migrants’ access to essential resources, including healthcare [17].

### Mental healthcare for migrants in Germany and in the federal state of Bavaria

As one of the leading destination countries in Europe, Germany has accommodated a considerable number of migrants, including a large proportion of refugees and economic migrants [18, 19]. However, research on mental disorders within this diverse population remains limited [20, 21]. Existing studies on specific migrant populations such as elderly migrants [22] or asylum seekers and refugees [21, 23, 24] indicate an increased burden of conditions like depression and PTSD.

The German healthcare system includes statutory health insurance covering 90% of the population and private health insurance covering the remaining 10%. The mandatory insurance requirement applies for nearly all migrants in Germany [25, 26]. However, there are differences for specific migrant groups. For example, the health insurance status of international students in Germany varies depending on agreements between their home country and the German healthcare system, requirements of their academic program, and whether they have statutory or private insurance. As a result, their coverage may not fully include essential services such as psychotherapy [27]. Asylum seekers on the other hand, are not included in the regular insurance system, and their healthcare access differs across Germany’s 16 German federal states [24, 28].

In Bavaria, a state in the south-east of Germany, asylum seekers must seek approval from the relevant authorities before every doctor’s visit to obtain a treatment certificate. Their healthcare coverage is initially limited to acute conditions, including psychiatric services, but access to psychotherapy is largely restricted. Instead, psychotherapeutic care is often provided at specialized psychosocial treatment centres [24]. Other migrant groups also encounter barriers to accessing mental healthcare in Germany, leading to their underrepresentation in inpatient psychiatric and psychotherapeutic facilities [21, 29].

Guidelines [30] and position papers [31] were published to improve mental healthcare for migrants, emphasizing early detection of mental health problems in migrants, migrant sensitivity in healthcare, the availability of interpreters, and better information for migrants. More recent research highlights the need for greater cultural openness within the German healthcare system but also points out that too few of these recommendations have been implemented [29, 32]. Studies focusing on asylum seekers highlight the negative consequences of the existing restrictive healthcare policies [28, 33].

Despite bearing a high burden of mental health problems compared to the general population [11, 22], migrants in Germany experience a variety of barriers impeding their access to mental health services [16, 28, 34]. Given that mental health and access to mental healthcare are essential human rights [11, 35, 36], ensuring equitable access for all migrants in Germany is both a legal and ethical imperative. To achieve this, creating action-oriented strategies is necessary, particularly since previous guidelines have not incorporated migrants’ viewpoints.

The aim of this study was to explore the determinants influencing migrants’ access to mental healthcare from the perspectives of migrants and health professionals in Munich, Bavaria, and to provide the foundation for the development of comprehensive recommendations for actions to improve migrants’ access to mental healthcare in Munich and beyond.

## Methods

### Study design

**Fig. 1:**
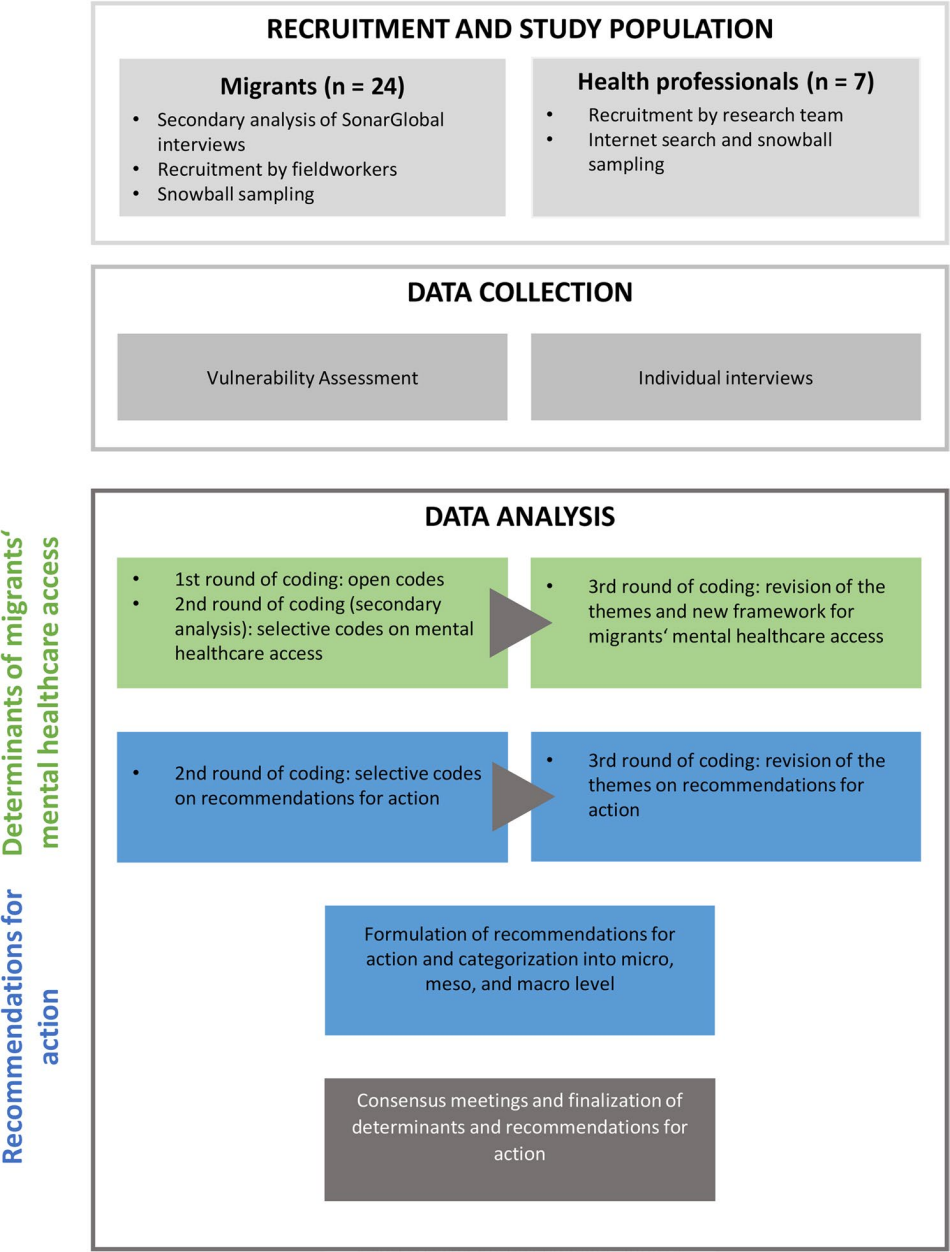
Overview of the methodology of the study

This qualitative study included the perspectives of two participant groups: migrants and health professionals (Fig. 1). The migrant participants were involved in 2021 as part of the German component of the SonarGlobal project, a five-country European Union initiative investigating the impact of the COVID-19 pandemic on vulnerability mechanisms using a Vulnerability Assessment Tool [37]. For the present study, a secondary analysis of the qualitative data was carried out. The use of qualitative secondary analysis is well established in health research in general and more specifically in studies involving migrants [38–40]. Aligned with the open data movement, it is increasingly encouraged to maximise the scientific value of existing datasets, saving time, costs, and resources [38, 40, 41]. Qualitative secondary analysis is particularly suitable for research on sensitive topics and involving groups that are difficult to reach, reducing the burden on participants [38, 41, 42]. A requirement is that the research questions are appropriate for the available data [42, 43], though they can differ from those guiding the primary data collection [40, 43]. In the present study, the broad scope of the SonarGlobal interviews and ZÖ’s contextual knowledge as the primary researcher [44], supported the data’s suitability for addressing the research questions in a secondary analysis. The analysis was guided by an intersectional perspective, recognizing that interconnected inequalities shape social structures and experiences [45]. Accordingly, the migrant participants were selected based on three groups with different mechanisms of vulnerability: (1) refugees, (2) LGBTQ+ individuals, and (3) international students. To complement the findings and explore potential recommendations for action, additional interviews with health professionals were conducted in 2023.

Access to mental healthcare was explored using content analysis based on Levesque et al.’s framework [46], which conceptualized healthcare access through five patient-related (ability to perceive, the ability to seek, the ability to reach, the ability to pay and the ability to engage) and five provider-related (approachability, acceptability, availability, affordability and appropriateness) dimensions. The researchers summarized and schematized the results from both participant groups by employing an adapted version of the model by Levesque et al. in an iterative process. Furthermore, the data from the interviews with migrants and health professionals was used to develop recommendations for action to improve mental healthcare access for migrants. These were initially organized thematically and subsequently mapped onto the three dimensions of integrated care according to Valentijn et al.: the micro, meso and macro level [47].

### Study setting

The study took place in the metropolitan area of Munich, a city in Southern Germany with 1.604.935 [48] inhabitants and the capital of the state of Bavaria. Owing to its geographical proximity to the country’s south and east border, Munich is home to a large share of refugees who are accommodated in a variety of facilities, including state and local refugee shelters as well as arrival centres [49]. In addition, Munich has a robust economy, a sizable employment market and three internationally recognized universities, making it an attractive destination for many migrants including international students. With 18.5% of the population being Germans with a migration background and 30.1% being foreign nationals without German citizenship, Munich is a diverse and multicultural city [50]. Munich thus plays a significant role in shaping German asylum policies and delivers various forms of aid to migrants, including one of Bavaria’s few psychosocial treatment centres [51]. More than half of the social organizations and initiatives receiving financial support from the city are related to migration [52].

### Recruitment and study population

#### Migrant participants

Participants from the SonarGlobal Project were reviewed for secondary analysis as part of the current study. We followed the recommendation published by Chatfield [41] to select a purposeful subsample that is best suited to answering the research question while reducing time and resource demands. As secondary analysis is particularly valuable for accessing perspectives of individuals who are otherwise hard to reach, they advise including participants who are usually underrepresented in regard of socio-demographic characteristics [41]. Of the 82 people aged 18 and above reached in the project, 24 people from three migrant groups who were living in the Munich metropolitan area were included in the present study. The three groups were selected based on their potential increased risk of mental health problems due to the intersection of vulnerability mechanisms identified in the primary study. First, refugees who have lived at least two months in refugee shelters in Germany within the last five years were included to represent the challenges encountered during various stages of migration and precarious living conditions. Second, to highlight the intersection of migration and gender-based discrimination, people who identified themselves as both LGBTQ + and migrants were invited to participate. The third group consisted of international students based on their report of at least one vulnerability factor, such as precarious housing, severe financial insecurity, a lack of social support, or a higher risk of discrimination because of their appearance. Participants who did not demonstrate these intersectional vulnerability mechanisms were not included in the secondary analysis.

The recruitment of the 24 participants in SonarGlobal was supported by three fieldworkers, all of whom were migrant students trained in qualitative methodologies. One of them was a member of an organization supporting asylum seekers in refugee shelters and also part of a Muslim community. Another resided in an international dormitory housing and was enrolled in a master’s program primarily composed of international students. The third was actively engaged in multiple LGBTQ+ associations and was a dedicated advocate for LGBTQ+ rights. The sampling process was guided by the principle of purposeful sampling. Each of the three fieldworkers identified individuals from their networks whom they expected to provide rich information while also experiencing multiple forms of vulnerability. The researchers (ZÖ and MC) and the fieldworkers discussed the suitability of these individuals for the study in dedicated meetings. The fieldworkers then invited eligible participants for interviews. Additionally, they employed snowball sampling to further expand the participant pool. Special attention was given to ensuring variation in terms of age, gender, and ethnicity among the participants. Due to the available language skills of the researchers and fieldworkers, the study group was limited to people who could speak German, English, Turkish, or Farsi.

#### Health professional participants

The study included mental health professionals alongside migrants. An internet search was conducted to identify and list psychotherapists, psychiatrists, and family physicians in Munich who work in institutions that only serve refugees, provide services in languages other than German, and/or work in areas with a high migrant population. Further, the search was broadened by including contacts through snowball sampling. Suitable persons were contacted via email and invited to participate in individual interviews. No one declined to participate. Seven interviews were conducted until data saturation was reached, which was determined when no new contents emerged and participants only elaborated on previously identified categories in the last two interviews.

### Data collection

#### Migrant participants

The Vulnerability Assessment Tool, consisting of a questionnaire and a semi-structured interview guide, was translated into German, Turkish and Persian and modified for use in Germany during the interviews with the SonarGlobal participants [37, 44]. The quantitative questionnaire covered various aspects, including demographic characteristics; household-related factors (such as type of dwelling, homeownership status, and household composition); income and expenses (such as annual income, financial support received, income sufficiency, healthcare insurance, the burden of healthcare expenses); employment (such as employment status, number of working days and hours, current occupation, and the likelihood of changes in these factors within six months); and existing health problems. It was used to determine vulnerability in the primary study. In the present study, only the qualitative component was reanalysed: The semi-structured interview form included questions exploring participants’ perceptions of health, both in general and in relation to their own circumstances, as well as their views on the determinants of health. It also addressed their assessment of the impact, if any, of their chronic health conditions on their daily lives, the sources of information they rely on regarding health and healthcare services, the support they receive from official institutions and social networks, their experiences with the German healthcare system, and their perceptions of equality within their neighbourhood and in access to healthcare.

The interviews were conducted by the three fieldworkers. They received training and ongoing support to use the Vulnerability Assessment Tool. Eighteen interviews were conducted face-to-face, while six were done online. The interviews took place in private settings, with only the interviewer and interviewee present. Due to the COVID-19 pandemic, infection control measures such as masks were used in face-to-face interviews. Of the interviews, ten were conducted in English, eight in Persian, and six in German. The Persian interviews were subsequently translated into English by the interviewer, whose first language was Persian. All interviews took place between April and May 2021.

#### Health professional participants

After the secondary analysis of the migrant interviews, individual semi-structured interviews with health professionals were done by SB and ZÖ in German. When conducting the interviews an iterative approach was chosen for several reason: (1) to avoid lengthy repetition covering the same topics in each interview, (2) to provide a clear structure to ensure that the discussion centred on recommendations rather than problem description, (3) to help validate prior findings, and (4) to enable participants to add new topics. Each interview began with the health professionals introducing themselves, their professional backgrounds and experiences with migrant patients. Following this, they received a 10-minute presentation of the results of the data analysis of migrant interviews, using a draft of the access framework developed by the researchers specifically for the mental health of migrants. They were requested for additions and comments on the determinants of access. In the second part of the health professionals’ interviews, recommendations for action were specifically questioned. This part of their interviews started with the presentation of the categorized recommendations that the authors derived from the analysis of migrant interviews and, where applicable, from the previous interview with health professionals. Then the interviewee was asked to comment the named recommendations and to add new ones. If necessary, the determinants previously discussed were used as thematic guidance. Each interview with a health professional was analysed before the next interview. Furthermore, questions on specific issues or migrant groups were posed to the health professionals based on their background and expertise. The interviews lasted approximately 45 min each and took place between February and May of 2023. Three of them were conducted through video chat, two at the health professional’s working location, and two at the researcher’s office.

### Data analysis

All coding was done in a three-part iterative process applying the content analysis approach based on Mayring and Fenzl [53] by using the software MAXQDA.

#### Migrant participants

The interviews with migrants were transcribed as verbatim transcripts by the respective interviewer. The open codes were developed by ZÖ and MC for the entire dataset of the German SonarGlobal project. The coding process incorporated the field workers’ notes from the interviews. SB and ZÖ reviewed the dataset of the migrants of the Sonar Global project, extracting open codes and data related to mental healthcare through mutual agreement. In the second round, SB refined the list of codes, and established sub-codes. In consultation with ZÖ, the codes and sub-codes were further categorized.

The researchers then discussed the categories related to determinants of access to mental healthcare in considering Levesque et al.’s access framework, grouped these categories under themes and visualised their connections. This newly designed framework is an adaption of Levesque et al.’s model that focuses on migrants’ access to mental healthcare. An iterative process was used to assess completeness of this framework by discussing the determinants in the interviews with health professionals.

Although no specific questions were asked about recommendations for action in the interviews with migrants, the research team observed that participants nonetheless expressed desires and suggestions for strengthening their access to mental health services. SB and ZÖ reanalysed the data with this perspective. SB reviewed the codes within the existing coding structure and added new codes when necessary. Together with ZÖ, relevant codes to reflect participants’ recommendations were revised and categorized.

#### Health professional participants

The interviews with health professionals were transcribed and coded immediately after they were conducted. SB categorized the transcripts into the present code and category system of the interviews with migrants so that the determinants of migrant’s mental healthcare access and the recommendations for action could be further refined. In addition to the existing ones, new codes and categories were added as needed and discussed together with ZÖ. By progressively adapting the coding structure, insights gained from earlier interviews were integrated into subsequent interviews. Hence the categories were developed in an iterative process and revised in repeated consensus meetings.

The analysis of all statements proposed by the participants to enhance mental healthcare access resulted in 17 recommendations across five themes. To illustrate the recommendations in an actionable way and to facilitate their implementation in the healthcare system the research team then established three recommendation domains adopting Valentjin et al.’s approach, which classifies integrated care into macro, meso, and micro levels [47]. They considered recommendations regarding structural vulnerability and causes of inequality at the macro level, recommendations on financing, delivery, and coordination of health services at the meso level, and measures to enhance access and boost resilience of individuals or groups at the micro level.

For validity, all results were discussed by the whole research team and presented to an additional psychiatrist, who has migrants as patients, and a professional in public mental health. Collaboratively, the framework of migrants’ mental healthcare access and the recommendations for action were finalized. The list of recommendations was distributed to the interviewed health professionals, but no major changes were necessary following their review. The migrants who participated in the study were invited to a follow-up meeting to discuss the findings. However, they did not express interest in attending, as their circumstances and priorities had changed over time.

The participants’ perspectives were exemplified in each category using direct quotes. SB translated the German quotes into English, maintaining the original words and syntax, even if it required translating grammatical mistakes. Afterwards, the original quotes and their translations were examined with ZÖ. Because all Persian interviews had already been translated into English, there was no need to translate these quotes.

### Quality assurance measures

The criteria of credibility, transferability, dependability, confirmability, and reflexivity guided the quality assurance of this qualitative study [54, 55]. Credibility was strengthened by including both migrants and health professionals as participants. Migrant participants were selected from an intersectional perspective, representing three groups with different vulnerability mechanisms. They were recruited and interviewed by fieldworkers who were closely connected to the respective communities and trained in qualitative methods. The interviews with health professionals from different disciplines were conducted and coded by the authors experienced in qualitative research. The diverse composition of participants further contributed to the transferability. Transferability was also supported by guiding the analysis and reporting of access determinants and recommendations through two conceptual frameworks. Dependability was improved by a two-step eligibility check for migrant participants by fieldworkers and researchers, as well as through independent coding by SB and ZÖ, followed by evaluation by MC and CJ. Confirmability was supported through the iterative interviews with health professionals, which allowed earlier insights to be revisited and refined, and through iterative analysis with validation meetings across the research team. External validation was provided by the additional experts, who reviewed the study’s results again.

Reflexivity was maintained throughout all stages of the research, with the authors continuously reflecting on their own positionalities and potential biases. All authors are white, cisgender women trained in public health. ZÖ, CJ, and MC are experienced researchers in healthcare in Germany, working with health professionals and structurally marginalized populations. ZÖ specialises in migration health, is a migrant herself and mother of an international student. SB also has close contact with international students through her academic program. Although the authors do not experience the same structural vulnerabilities as the participating migrants, they repeatedly discussed how their personal experiences and assumptions may have impacted the data analysis and interpretation of findings. Particular attention was paid to highlighting participant perspectives while mitigating bias resulting from the authors’ identities.

### Ethical considerations

The study was conducted in accordance with the ethical standards of the Declaration of Helsinki [56]. Ethical approval was obtained from the Ethics Committee of the Medical Faculty of the LMU Munich, reference number 21–0244. All participants provided written informed consent prior to participation.

## Results

### Descriptive information on the study population

Of the 24 participating migrants, 10 identified as female, 12 as male and two as gender nonconforming. There were 11 students, 12 refugees, three of which were both students and refugees, and four individuals identifying as LGBTQ+. Their ages ranged from 20 to 68 years. Two participants were born in Germany, while the others had been living in Germany for between one and seven years. When asked about their national identity, participants named 16 different affiliations, with Afghanistan (*n* = 8) being the most common. Almost all participants were covered by statutory health insurance, although refugee participants described a period without health insurance upon first arriving in Germany. One student was insured through foreign family insurance recognized in Germany. Another participant had private health insurance. An overview of all participating migrants can be found in Table 1.

**Table 1:** Characteristics of the study population – Selected migrant participants from Sonar Global

Characteristics of migrant participants (*n* = 24)		*n* (%)
Group*	Student	11 (46)
	Refugee	12 (50)
	LGBTQ+	4 (17)
Age group†	20–29	15 (63)
	30–39	6 (25)
	40–49	1 (4)
	≥ 50	1 (4)
Gender	Female	10 (42)
	Male	12 (50)
	Gender nonconforming	2 (8)
Health insurance	Statutory	22 (92)
	Other	2 (8)
Self-identified national identity‡	Afghanistan	8 (30)
	Syria	3 (13)
	Iran	2 (8)
	America	1 (4)
	Bulgaria	1 (4)
	Colombia	1 (4)
	Eritrea	1 (4)
	India	1 (4)
	Kenya	1 (4)
	Pakistan	1 (4)
	Romania	1 (4)
	Saudi Arabia	1 (4)
	Somalia	1 (4)
	South Korea	1 (4)
	Turkey	1 (4)
	Uganda	1 (4)
Years spent in Germany	1–4 years	10 (42)
	5–7 years	12 (50)
	Born in Germany	2 (8)

* Three participants were both students and refugees

† One participant did not disclose age

‡ Two participants mentioned two nationalities

As can be seen in Table 2, the health professionals all had varied work experiences and patient groups. Five of them were psychological psychotherapists, one was a family physician, and one was a psychiatric specialist. One of the health professionals worked in an acute psychiatry clinic for migrant youth, two in their own practice, one in a refugee-specific organization, and three in a university clinic. Three had refugees as patients and all of them had treated international students or LGBTQ+ persons. Only the family doctor worked in a rural area; the others were in the centre of Munich. Four health professionals received academic training in public mental health. Two of them were researching the role of primary care in mental healthcare access with one specialising in psychoeducation, while another one had publications on male gender roles in Muslim families. Three of the seven health professionals interviewed identified as migrants, while only one was male. The expert who attended the data analysis validation meeting was a female psychiatrist who had migrated and worked at a university.

**Table 2:** Characteristics of the study population – Health professionals experienced in mental healthcare

Health Professional	Migrant	Gender	Expertise
*H01*	Yes	Female	Researcher in public mental health, youth psychotherapist in training
*H02*	Yes	Male	Psychological psychotherapist, own practice
*H03*	No	Female	Psychological psychotherapist, researcher in public mental health, working experience with refugees in psychiatry
*H04*	Yes	Female	Psychological psychotherapist, researcher in public mental health and the role of primary care
*H05*	No	Female	Psychological psychotherapist, working with refugees in a psychosocial treatment centre
*H06*	No	Female	Family physician in a rural area where many migrants and refugees are living
*H07*	No	Female	Medical specialist for psychiatry and psychotherapy, working in psychiatry, researcher in public mental health

### Determinants of migrants’ mental healthcare access

**Fig. 2:**
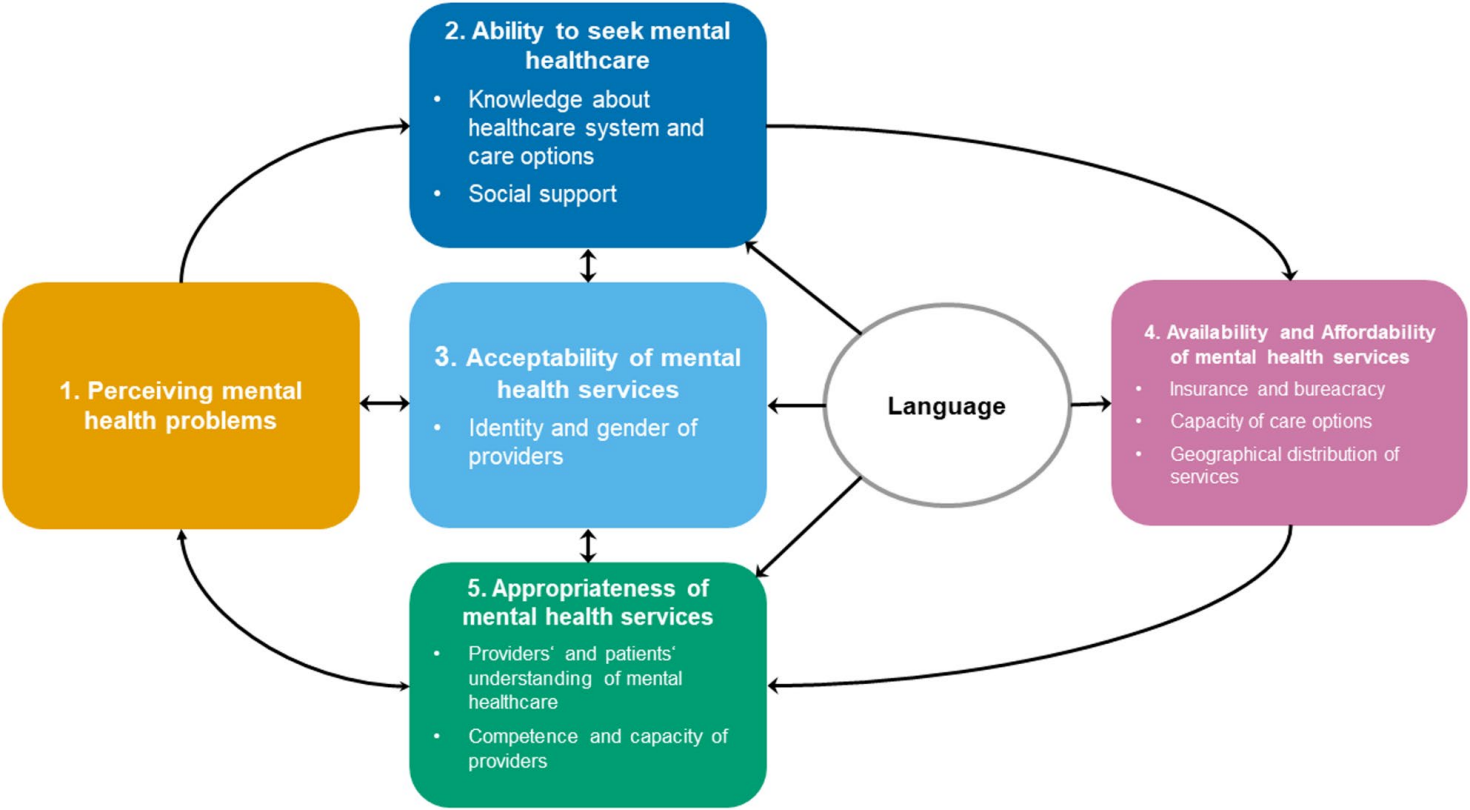
Adapted framework for migrants’ mental healthcare access (own presentation) based on Levesque et al. [46]

Based on the analysed interviews, the determinants of migrants’ mental healthcare access were categorized into five themes. Their relationship to each other is shown in Fig. 2.

#### Dimension 1. Perceiving mental health problems

The participating migrants’ perception of mental health varied widely. Those under the age of 30 reported mental health as a component of overall health and correlated their own mental health with physical symptoms such as weight loss or gain, migraine, hypertension, rashes, or immunological diseases.

“Health is two different things: physical health and mental health. […] And for mental health, I think that would require a lot more conscious effort, especially if you have any underlying issues that you still haven’t identified. […]. Just because they’re physically healthy doesn’t mean you’re mentally healthy and vice versa.” – International student, male, 23 years old.

Particularly older migrants and female refugees defined health in terms of physical well-being and did not directly address the topic mental health. Most of the health professionals stated that many of their migrant patients were less aware of their mental symptoms and thus often do not recognize when they need help. According to them, this can result in mental disorders manifesting in physical symptoms, making it difficult to detect them.

“How do illnesses manifest themselves in different cultural circles? For example, there are cultures where depression classically manifests itself with physical complaints. That they have very strong whole-body pain, fatigue, exhaustion. And then you have to pay more attention to these symptoms.” – H07.

Some male migrants described stigma about mental health problems. Among them, one from Romania and one from Afghanistan reported that mental health problems are considered taboo in their home country. A refugee from Syria expressed feelings of shame regarding his own mental health problems. They all saw the stigma associated with mental health as a reason why they or others would not talk about their struggles. Men were less likely to admit their mental health problems, according to two of this group.

“In [country] it’s really hard to get any advice about the depression. So, they tried to hide it. […] If I am depressed, I try to hide it to my parents or my friends. I try to overcome it by myself.” - International student, male, 27 years old.

One psychiatrist, the psychotherapist with his own practice and one psychotherapist working in a primary care stated that mental disorders are stigmatized, particularly among some migrant groups. Therefore, many do not acknowledge having problems and needing external help, which is especially true for men owing to their gender role of being “strong”, as noted by all health professionals who were migrants themselves.

“Actually, there are problems, they seek help, but there is a stigma. I remember last year a suicide […] of a teenager. A year ago, she has not expressed suicidal thoughts, but then one day we heard she is already dead […]. Then family needed many supports because in one day they have lost their daughter, but family has said […] no, they have not said anything […]. No, they have not accepted any therapy offer.” – H01.

#### Dimension 2. Ability to seek mental healthcare

##### Knowledge about healthcare system and care options

A small number of the participating migrants mentioned mental health treatment options that they were aware of after being asked if they knew where they could get medical support. However, one of the LGBTQ+ group thought psychotherapy was prohibitively expensive and was unaware that it is covered for those who are part of the regular health insurance system in Germany. The health professionals agreed that the knowledge about the German healthcare system has a major influence on migrant’s ability to seek help. The family physician and one psychotherapist working in a primary care project emphasized that too little guidance is provided to migrants on insurance and cost coverage of psychotherapeutic services. Additionally, they said that there was a lack of information about different treatment options. According to the psychiatrist and the psychotherapist with research experience in primary care and psychoeducation, the knowledge was often further restricted by a language barrier. A psychotherapist describes the situation of her patients in refugee shelters as follows:

“The knowledge about treatment services. Yeah, I think that’s so random still, whether they learn about something or not. If there is someone in a shelter who is well-informed, that person will pass on the information. But if they are in a shelter where people don’t know about it, then they won’t receive the information. Some […] search for it themselves on the internet, while others, especially those who don’t speak the language […], struggle.” – H03.

##### Social support

Both participant groups showed that having a social network and support system could have a significant influence on migrants’ ability to seek care options. Five migrants from all three groups described situations in which acquaintances or members of their community assisted them with healthcare issues. This could help in overcoming communication problems during doctor’s visits and making it easier to locate appropriate healthcare services. Two of the three participating migrants receiving psychological treatment stated that they found their therapist through another migrant’s recommendation.

“I have a colleague who is a psychologist, she also does counselling. Then I asked her and she gave me this advice. I should get myself a psychologist. I couldn’t with her, so do that, because I know her. So find a colleague of hers.” - Refugee, male, 35 years old.

The psychotherapist with research experience in primary care and psychoeducation working with refugees also shared her experience of how a personal contact with a refugee home led to her psychiatric clinic accepting multiple patients from the facility. The statements of the participants indicated that organizations and volunteers could also provide support in seeking care when there was no social network. Some refugees reported receiving support from social workers in their shelters, which helped in a variety of situations, such as assistance with insurance issues and doctor’s appointments, as well as support with translation or childcare. However, this was not the case for most participating migrants, as they reported receiving no assistance. Some of the refugees among them were aware of social organizations, but they either were not offered help at all or only got support for issues unrelated to their mental health. One refugee described that the capacity of social workers was not sufficient:

“My container is about 300, 400 lived. After that, three persons is not enough. That’s not enough staff for 300 […]. Then none comes fast to explain which document, or what e.g., job center wants or e.g., in social. Then not so good explanation. They say, then this address you must go, then gone.” - Refugee, female, 32 years old.

Several participating migrants added that because of limitations imposed during the COVID-19 pandemic, support from organizations had disappeared. In addition, language barriers were a commonly mentioned impediment to social assistance, which was endorsed by the health professionals. A psychotherapist working with refugees emphasized the lack of support in refugee shelters, particularly for mental health problems. Moreover, the family physician and the psychotherapist working in a psychosocial treatment centre stated that support is especially important for women, who are frequently hindered from accessing healthcare due to the responsibilities for their children. The family physician provided the example below.

“Just a young patient who is here with a disabled child and a school child, so with a disabled kindergarten child, alone from Lebanon now here. There is family, living at a distance, maybe 10 km, but she lives here alone in a council flat, or in a room, and does not speak the language.” – H06.

#### Dimension 3. Acceptability of mental health services

##### Identity and gender of providers

Prejudice on the side of both migrants and health professionals based on the social identity of the other functioned as a barrier to professionals’ acceptability. Many refugees from Arabian countries and migrants who identified as black reported a lack of trust in physicians and inhibitions about confiding in them due to fear of not being understood. Especially those persons and some of the LGBTQ+ group described discriminating, racist encounters in the medical setting, which is why they would disregard accessible medical professionals in favour of those with the same cultural background or ethnicity.

“[…] the doctor’s offices where they treated me as if I did not understand German. Like a little child, you then also talk like a little child. Or slowly speaking German. So, this, I’m black, which automatically I don’t understand German.” - LGBTQ+, female, 27 years old.

Many health professionals agreed that migrants often preferred professionals with the same cultural background because they were afraid of being misunderstood, or even of being judged. Such fears were frequently exacerbated by a language barrier. The health professionals saw reservations on both sides: According to the psychotherapists who are migrants themselves, some of their migrant patients had unfounded prejudices against therapists when they do not have the same origin. On the other hand, according to the health professionals working with refugees, the concerns are reasonable, as some German health professionals feel overwhelmed by migrant patients and may refuse their treatment out of fear of doing something wrong. According to them, some health professionals had difficulties to empathize with their patient’s different backgrounds. Their patients reported negative experiences or even discriminatory prejudices.

“It’s a bit absurd, because all psychotherapeutic colleagues should actually be able to deal with trauma. […] But for some reason, many colleagues don’t dare to: trauma, anything can go wrong. Not true, but worry. And then actually this combination of ‘I have a migration background’. Then Germans sometimes don’t dare to because they think: I’m missing something.” – H05.

Almost all health professionals added that both men and women depending on their cultural background may find it difficult to see a doctor of the opposite sex: Men were more likely to question the competence of female healthcare providers, whereas women found it harder to open up to men.

“There are sometimes problems: men-women communication. That perhaps men from perhaps patriarchal cultures had difficulties with female doctors and then somehow try to meet them and then perhaps to also always talk with a male colleague.” – H07.

#### Dimension 4. Availability and affordability of mental health services

##### Insurance and bureaucracy

The health professionals reported that many migrants among their patients had trouble arranging medical appointments, mainly owing to the language barrier. The refugees experienced the most challenges. Several of them described major bureaucratic hurdles they were confronted with, because they did not have a health insurance, making it very difficult to make appointments or obtain reimbursement for drug costs.

“The people who are supposed to go to the doctor […] they are supposed to pick up a health certificate from the asylum office […] and that takes time with the bureaucracy and it’s also such crap. What if they are sick? […] if they’re also depressed and also don’t get these things on their own […] then I don’t know.” - Refugee, male, 35 years old.

“I had an appointment, but I didn’t have my insurance paper. […] So like when I reached the lady, told her that I don’t have the insurance, but it’s going to come in three days. She told me to go back and take it. So then I left. One hour after, the doctor called me. He was like, you have an appointment. I said […] they told me to go back and bring an insurance, which I don’t have. […] So he was like, come back and get your treatment. You’ll bring the insurance paper.” - LGBTQ+, gender nonconforming, 30 years old.

According to the psychiatrist, family physician, and psychotherapist working at a psychosocial treatment centre, this not only limited patients’ ability to access healthcare, but also burdened health professionals with extra strain created by patients’ inquiries about bureaucratic matters.

“That would also be a classic thing in standard care: A refugee client comes in with five letters and first says: I don’t understand them. And then the person in regular care must first try to consider: No, that’s not important, that’s advertisement, ah there we have something from the health insurance.” – H05.

Based on the opinion of most of the health professionals, the inability to pay for medical services also primarily affected individuals lacking health insurance, and therefore especially refugees in the asylum seekers process. On the other side, all participating migrants with health insurance did not perceive the expenses associated with doctor visits and medication as an obstacle to receiving healthcare.

##### Capacity of care options

All health professionals highlighted the insufficient availability of psychotherapeutic or psychiatric treatment spaces. They indicated lengthy waiting periods, ranging from six months to two years.

“In the last two years, one and a half years, they are looking for therapy spot, counselling spot, there is nothing at all. And I know practices, they write in their website now: please don’t call us again in the next two years.” – H01.

One participating refugee currently undergoing psychotherapy shared his experience of waiting six months to secure a therapy spot. This was acknowledged by the health professionals as a fundamental issue that affected all individuals in Germany. However, they stated that it posed even greater challenges for migrants due to limited options in other languages and the excessive demand on specialized services, such as psychosocial counselling for refugees. The health professionals added that capacity was also lowered dramatically as a result of the COVID-19 pandemic.

“The question is, are there offers, and especially in the native language. So psychotherapists who perhaps do therapy in English, there are already several. But there are very few in Farsi or other languages. And there the rush is big, of course, and the waiting times get then actually, too […] they are then very quickly overrun, if there are very many…so, if there is a great need.” – H03.

##### Geographical distribution of services

Five health professionals highlighted transportation and the location of treatment facilities as a component of availability. They noted a disparity in therapy spots between urban and rural areas, with fewer suitable options for migrants in rural regions. While rural networking among doctors may offer advantages, according to the family physician, long distances to care facilities and inadequate transportation possibilities posed challenges for migrants. Most of the participating migrants reported no such problems owing to residing in the urban region of Munich, but refugees staying in isolated places experienced considerable difficulties.

“I have a client, it is not that far away, he comes here in an hour […] But where he is, there is nothing else. […] he speaks French and English, so you could expect him to find someone. But he has no chance, and he is highly depressive and severely dissociative. […]. So, that means, it simply needs to be spread much more widely.” – H05.

#### Dimension 5. Appropriateness of mental health services

##### Health professionals’ and patients’ understanding of mental healthcare

The health professionals pointed out that the treatment of mental disorders can be influenced by how these disorders are defined, noting that the understanding varies across cultures. They explained that German approaches emphasize individual responsibility and active participation in recovery. In contrast, they observed that some of their migrant patients perceive mental health problems as an external force. According to them, this perspective affected their therapeutic approach as these patients tended to adopt a more passive role during treatment.

“Before they come to us, they go to their religious places, with amulets, incense […]. They come, they have the expectation: […] Someone made me sick. Evil eye has made me sick. And I come to you, and you have to make me well again, because you are an expert. Because you’re a doctor or a psychologist. So I don’t have to do anything for it.” – H02.

If therapists were not prepared to take this into account, these differing beliefs and expectations could potentially clash, creating challenges for both the patient and the health professional. Instead, they emphasized that treatment of mental disorders should always be tailored to individuals’ specific needs without generalizing, and that therapists should be able to resonate with all patients, regardless of their background. Many female students and refugees as well as almost all LGBTQ+ migrants also expressed the desire that their doctors would empathise more with their perspective. One LGBTQ+ person wished for more alternative medicine, including spiritual components such as shamans.

“Because of course my idea of treatment goes in a completely different direction, when I, as a German, say: post-traumatic stress disorder, of course, trauma. […] And my client thinks: Actually, I am bewitched. Then I don’t need to come up with a trauma confrontation.” – H05.

##### Competence and capacity of health professionals

Provider competence and patient satisfaction emerged as important factors determining the appropriateness of care. Two women and one gender nonconforming migrant reported being misdiagnosed or not being taken seriously. One psychotherapist with research in primary care working with refugees emphasized the problem of undiagnosed psychoses among migrants. The other health professional working on primary care research mentioned that many migrant patients feel a lack of understanding. According to both migrants and health professionals, the quality of treatment was also heavily influenced by whether the health professional and the patient could communicate in the same language. However, an equal number of LGBTQ+ persons and refugees had positive experiences with health professionals, which often led to a trusting relationship. One of the named psychotherapists confirmed that this was mostly evident with their primary care physicians. According to the psychotherapist working in a psychosocial treatment centre, the next generation of psychotherapists is becoming more adept at treating people from diverse backgrounds. One participating migrant from the LGBTQ+ group described her psychotherapist as follows:

“It was important that I spoke to her sooner because she could understand my reasons, reason, or cause for my depression rather better than others.” - LGBTQ+, female, 27 years old.

The psychiatrist, general practitioner and one psychotherapist additionally emphasized that the amount of time available for patients was a crucial factor in their competence of providing appropriate care. They said that some patients with migration experience required more time compared to other patients due to their specific needs, such as dealing with bureaucracy and communication challenges. The psychotherapist working at the psychosocial treatment centre emphasized that, in contrast to standard care, she had enough time to engage with her patients’ perspectives in depth during therapy:

“One factor we have, is time. The regular care system doesn’t have that. So I can just take three hours of therapy to find out exactly those kinds of things. If someone is sitting with a family doctor, it’s difficult. He has a maximum of a quarter of an hour.” – H05.

### Recommendations for action to improve migrants’ mental healthcare access

**Fig. 3:**
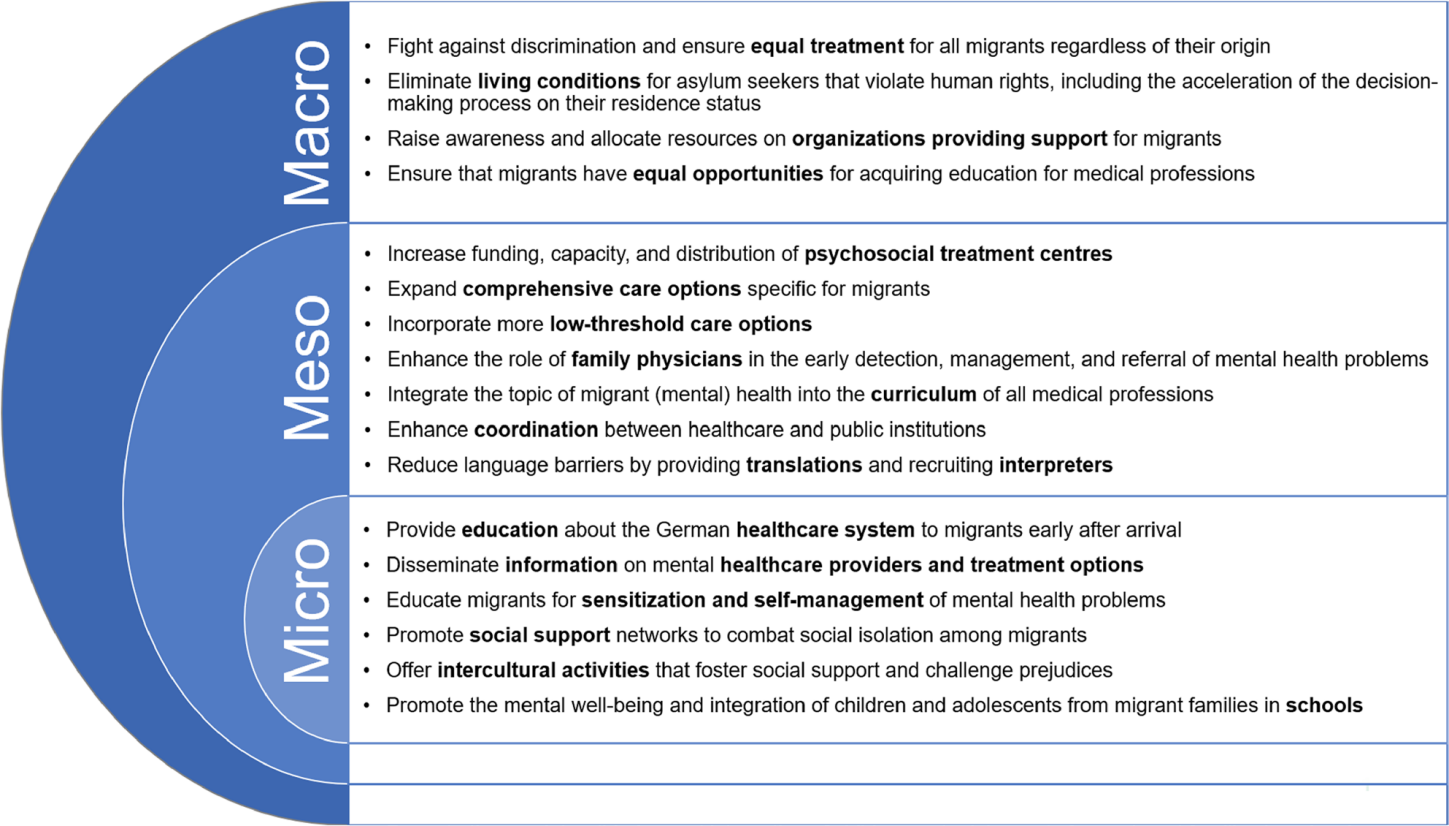
Recommendations for action to improve migrant’s mental healthcare access on macro, meso, and micro level

The iterative analysis of the interviews with migrants and health professionals resulted in a list of 17 recommendations under five themes aimed at enhancing the accessibility of mental healthcare services for migrants. These consisted of: (1) Structure, Organization, and Funding of Healthcare; (2) Healthcare Providers; (3) Adaptation to a New Healthcare System; (4) Social and Organizational Support; (5) Determinants of Health (see Supplementary Table 1, Additional File 1). The first theme related to the structural components of the healthcare system. Theme 2 focused on healthcare providers and their training. The recommendations of Theme 3 aimed to assist migrants in navigating a new healthcare system and finding the right care options. All recommendations about external support for migrants were summarized in Theme 4. Theme 5 comprised all recommendations that were associated with improvements in migrants’ mental health. Figure 3 shows the recommendations for action after they have been categorised into the three dimensions of integrated care following the approach of Valentijn et al. [47].

#### Macro level

At macro level, the health professionals working with refugees and the ones who were migrants themselves stressed the need for equitable societal and political responses to all migrants, irrespective of origin. They observed that refugees from Ukraine were often received and supported differently compared to those from Syria or Afghanistan. Fighting discrimination was also a recurring demand in interviews with migrants from Arabian countries, as well as those who identified as black or/and LGBTQ+.

“To fight racism fundamentally, first of all. To eliminate discrimination. […] Yes, to eliminate unequal treatment. Well, that requires a lot of change in society. But first of all, the laws need to be changed. […]. Yes, we are in a state-run country. So the state also has to change something.” - Refugee, male, 35 years old.

Many refugees pointed out how important a faster decision on their residency status in the asylum application process is for their mental health. This was also stressed by all health professionals working with refugees, adding that it would not only promote mental health, but also improve their mental healthcare access by facilitating a quicker integration into the regular healthcare system and providing earlier opportunity for migrants to work in the psychosocial sector themselves. According to the health professionals who were not migrants, equal opportunities in the education and training of medical professions would increase the representation of migrants among healthcare providers bridging language barriers and fostering better patient acceptance. A psychologist who migrated described how challenging it is for her to undergo training in Germany in order to practice as a therapist:

“It’s a very long journey in Germany. You first need to have a master’s degree, and then that takes 3–5 years. An insane program. It is long training program. […] We have to work many hours in therapy, 2400 hours as an intern.” – H01.

**“**So that the system changes in such a way that it might also become easier for people with a migration background to enter these professions. […] Most psychotherapists often come from very wealthy families, with academic parents, who had a lot of support and resources.” – H03.

The health professionals working with refugees emphasized the need for better housing conditions for asylum seekers. The health professionals and most participating migrants agreed that organizations providing support for migrants should be strengthened. One health professional with public health research experience stressed the significance of carrying out further research on the mental health of migrants. As no other health professionals or migrants mentioned this, it was not included in the analysis as a recommendation for action.

#### Meso level

The recommendations for action at the meso level mainly related to the coordination and collaboration among different organizations and healthcare providers. All health professionals advocated for better coordination between healthcare and public organizations. Thereby, they emphasized the important role of general practitioners and psychosocial treatment centres. The importance of both also emerged in the interviews with the migrants.

Most of the health professionals stressed including migrant health into the training of all medical professionals to provide culturally appropriate treatment. They emphasized the importance of being open to their patients’ perspectives rather than reinforcing stereotypes. The psychotherapist working at the psychosocial treatment centre described it as follows:

“So to say: Okay, you have this idea. […] What exactly does it look like? To explore that. And then I would place my concept next to it. Say: Okay, the Western psychological perspective might have this kind of explanation. That doesn’t mean one is better or worse […]. And then to say: Where does your explanatory model come from, where does mine come from, and can we find an overlap that allows us to say it’s still worth approaching it from there.” – H05.

All health professionals suggested low-threshold care options, such as self-help groups, or digital health applications. The psychiatrist and the psychotherapists conducting research in primary care additionally recommended comprehensive care options for migrants such as family therapies, body-awareness approaches, occupational therapies, or including language acquisition as a component. Some migrants from all three groups also expressed a desire for alternative care options. All migrants who did not speak German underlined the need of understandable healthcare services.

“More translators, yeah of course […]. I think language plays a big role, so if you don’t know the language, then you think everyone is racist or everyone is aggressive towards you, which is very normal. When you don’t know the language. If you know the language, then you communicate, then you can do it. Didn’t have any problems. You just felt like there was a problem.” - Refugee, male, 28 years old.

Equally, all health professionals agreed on the significance of interpreters and translations, including their cost coverage by insurance.

#### Micro level

On the micro level, the participants’ recommendations for action emphasized a person-centered approach, promoting continuous and individualized care to address the specific needs of patients. The family physician and three psychotherapists suggested conducting educational activities aimed at migrants to provide information about the German healthcare system. Many of the participating migrants, especially refugees and international students, whished for more information on available healthcare services. The health professionals additionally encouraged strengthening migrants’ mental health awareness. Their recommendations included the use of digital applications, with a particular focus on children and adolescents, as well as mental health education in schools. Additionally, most psychotherapists and the family physician proposed using regular check-ups as an opportunity to initiate conversations about mental health, and organizing dedicated informational events to raise awareness in refugee shelters or practices.

“That this is simply communicated very differently in public healthcare […]. As a leaflet: problems with mental disorders, with etc. That general practitioners are listed, locally. That, for example, reference is made to an organization that isn’t too far away. […] in printed form, or digital. […] Like a kind of address pool for all sorts of things. Whether it’s basic healthcare from general practitioners, or psychosocial counselling centres. […] Also for addiction issues, post-traumatic stress disorder or depression.” – H06.

In order to promote mental health, almost all health professionals and migrants emphasized the importance of social support. One refugee shared the positive impact that volunteering in his refugee shelter had on his own social network and mental health:

“They were engaging two days a week. So I was just waiting for the Mondays […], so I can have different, completely different feeling that I’m still alive. So I’m engaging in something. […] It had like positive psychological impact. […] And I started becoming part of their circle, […] so that’s quite, quite good experience. […] Everyone is different. Some people don’t want to do it, because it also gives you stress. People who have the same, like, they also share the same problems you have. But still, I think it’s good if you’re helpful, and that’s what I did. Just engaging in a volunteer group, make a social circle.” - Refugee, male, 28 years old.

Several health professionals recommended offering intercultural leisure activities to both foster social connections and combat racism. Besides, the health professionals who were migrants themselves recommended refocusing schools on fostering personal interests and linguistic development of children from migrant families to promote their mental health and social integration.

## Discussion

Access to mental healthcare is a fundamental human right that should be upheld for all migrants. This study explores the factors that determine access to this right in Munich, a Central European city and home to a wide range of migrant communities, through the perspectives of both migrants and health professionals. While our focus on three specific migrant groups—international students, refugees, and LGBTQ+ individuals—may limit the generalizability of the findings, this selection was a deliberate methodological choice. These participants were drawn from our previous research (SonarGlobal), where they had been identified as experiencing multiple layers of vulnerability. Their interviews were reanalysed with a particular focus on mental health. Our approach was guided by an intersectional perspective [45], which challenges essential understandings of identity that treat social categories as fixed and homogeneous. Instead, intersectionality emphasizes how different axes of inequality interconnect to shape both social structures and personal experiences. This perspective also informed our discussion on the role of culture in shaping access to mental healthcare.

The Levesque et al. framework, which is also commonly employed to conceptualize the access to mental healthcare for migrants [57, 58] was adapted for this particular group and mental health through the iterative process we followed. This modified model served as the foundation for a structured and comprehensive development of recommendations for action based on the interviews with both migrants and health professionals. The approach resulted in 17 recommendations aimed at improving migrants’ mental healthcare access, which provide the basis for the development of comprehensive and up-to-date recommendations for action.

### The framework for determinants of migrants’ access to mental health services

The framework developed in this study summarizes the key determinants identified through our findings. Unlike Levesque et al. [46], we did not draw a strict distinction between patient and provider perspectives. Rather, the progression of dimensions in our framework illustrates a shift from the patient side (such as the ability to perceive and the ability to seek) to the provider side (including acceptability, availability and affordability, and appropriateness), while also emphasizing their interconnections. We also combined availability and affordability into a single category, reflecting the German context: While statutory health insurance covers the majority of the population, including most migrants, asylum seekers face limited access [51]. For them, cost coverage emerged as a critical barrier, often depending on available support services.

While Levesque et al.‘s present access as a linear process, our findings point to a more circular and interconnected structure. Language, for instance, influenced nearly every dimension of access—from the ability to seek care and communicate with social organizations, to the acceptability of providers’ language skills, the appropriateness and perceived quality of care, and the availability of services in one’s native language. This reinforces the need to understand access as a complex, overlapping process, particularly in the case of migrants.

Our findings on the dimension ‘Perceiving mental health problems’ are comparable to those reported in Western nations, including Germany, indicating the effect of stigma [13–15, 59–61]. External stigmas, such as those based on gender roles, can cause migrants to worry about their social standing, preventing them from recognizing their mental health problems [13, 59, 61]. Culture shapes mental health understanding, as many authors have noted [13, 61, 62]. Migrants may recognize their mental health needs, but their understanding of mental health problems and preferred forms of support may differ from Western mental health paradigms, leading them to seek help through culturally familiar pathways [13–15, 63]. Other authors advocated for integrating non-Western approaches into care, rather than excluding them [64, 65].

According to our findings, the knowledge of treatment options and the healthcare system was particularly important for newly arrived migrants, such as international students or refugees. The need for accessible and targeted information is widely acknowledged in existing recommendations [61, 66]. In line with current guidelines [67–69], our study further emphasizes early detection of mental health problems, either through organizational support or raising awareness among healthcare workers. While Levesque et al. [46] conceptualize social support as one element within the ability to seek, our findings suggest that it plays a far more central role in both access to mental healthcare and migrants’ mental health. Participants across all three groups in our study—international students, refugees, and LGBTQ+ individuals—consistently emphasized the significance of social networks not only in navigating the healthcare system but also in coping with mental health problems. This broader relevance of social support is also well-documented in existing research and guidelines, which frequently highlight its role in facilitating migrants’ integration and improving their mental health [12, 14, 30, 65, 67].

In contrast to other countries where insurance coverage can be a significant barrier to affordability for migrants [13, 59, 70], cost of health services was rarely an issue for the participating migrants in this study. However, this was not the case for the refugees during the asylum seeker procedure. As in previous research [29, 33, 61, 62], we found that their exclusion from regular insurance created access barriers, including bureaucracy [23, 61, 64]. Even though this issue did not emerge among the international students in this study, it may also affect those whose private insurance does not fully cover mental health services [27]. The study’s recommendations for addressing these issues include increasing funding for psychosocial treatment centres, improving collaboration between social organizations and the healthcare system, and implementing mental health prevention measures.

In Germany, where the capacity of the mental healthcare system is constrained and where waiting times for the general population are already long [71, 72], access to services for asylum seekers is even more restricted [23, 24]. Our findings confirm that institutional capacity is particularly limited among organizations that either provide services in multiple languages or rely on alternative funding sources outside the regular insurance system. In our study, geographical accessibility showed as a barrier for refugees who remain housed in remote refugee shelters, which is also acknowledged in existing literature [73]. To address this, our study recommends expanding low-threshold mental health services, echoing calls made by other researchers [13, 74, 75].

Several studies have shown that inadequate communication between healthcare providers and patients can lead to misdiagnoses and ineffective treatment [11, 14, 62, 67]. Language emerged as one of the central determinants of access in our study, not only shaping patients’ ability to seek care, but also influencing the access to information about treatment options, and in their trust in providers. Yet, challenges between patients and healthcare workers extend beyond language. Negative, and at times discriminatory, experiences stemming from health professionals’ lack of cultural sensitivity or competence can undermine trust in the healthcare system [13, 59]. Such instances were also reported by participants in our study, who described being treated unfairly due to country of origin, language proficiency, skin colour, gender identity, or sexual orientation.

Cultural competence is widely recognized as a key determinant in health professionals’ ability to address the mental health needs of migrants [11, 14, 69]. However, both the concept of culture and the idea of cultural competence remain subjects of critical debate. Culture is not a fixed or neutral category but a fluid and politically charged construct shaped by power relations and broader societal dynamics [76, 77], raising questions about who defines cultural “difference” and with what authority or intention [45]. These debates extend to where culture is located in relation to other dimensions of identity—such as gender, class, or trauma history—and how these categories intersect in shaping individual experiences [45]. Cultural categorization often involves the construction of hierarchies [45]. Broom and Kirby [78] critique the tendency to reduce cultural complexity—of both providers and patients—into simplified interpretative frameworks and advocate for more nuanced approaches that engage with the intersectional realities of individuals. Our findings reflect this critical stance. While both participant groups acknowledged that shared cultural norms may influence perceptions of mental health, they showed that access was more significantly shaped by the intersection of past experiences—including trauma—, risks of discrimination based on sexual identity, ethnicity, or legal status, and anxieties around settlement. Health professionals’ emphasis that patients should be viewed not as cultural representatives but as unique persons with mental health needs arising from complex, intersecting vulnerabilities aligns with a person-centred rather than a culture-centred approach.

### Recommendations for action at the macro, meso, and micro levels

The recommendations of this study correspond to interventions at the macro, meso, and micro levels of the healthcare system, in line with the framework of integrated care conceptualized by Valentijn et al. [47].

A key macro-level recommendation of this study is the urgent need for a healthcare system free from racism, discrimination, and resulting structural inequalities. This aligns with a substantial body of literature and policy guidelines identifying racism and intersectional discrimination as critical determinants of both mental health and access to healthcare [10, 13, 14, 29, 57, 66, 68, 79, 80]. In this light, our findings are far from surprising. The implications of this recommendation are particularly salient for individuals undergoing the asylum process, whose access to basic rights and healthcare is shaped by systemic barriers. Specifically, accelerating the determination of residency status [15, 33, 80] and improving living conditions in refugee shelters [62, 80, 81] have been widely advocated as essential to protect their mental health. Our findings indicate that such interventions at macro level are equally crucial for enhancing access. Both German and international research has stressed the need to dismantle restrictive policies that limit healthcare access for refugees and asylum seekers [3, 28, 33, 66, 82]. Bhugra et al. [65] further underscore that the failure to provide adequate interpretation services constitutes a form of indirect discrimination, reinforcing systemic inequalities in care provision.

Guidelines also called for expanding data collection and research to monitor migrants’ mental health and their access to services, and to introduce appropriate policies [65–68, 83]. In Germany, such efforts relate to the concept of ‘intercultural openness’ [84, 85], which is suggested to be recognized as a quality aspect of services [29, 86]. Although not included in this study’s recommendations for improving migrants’ mental healthcare access, as the health professionals’ emphasis was elsewhere due to their specialization of work, many aspects align with the results of this study, such as the promotion of employees with a migration background on the macro level [85].

In line with our findings at the meso level, many studies and guidelines called for better cooperation between social organizations and the health system, especially for refugees’ and asylum seekers’ care [15, 31, 61, 62, 66, 80, 82]. It can assist in overcoming the numerous bureaucratic barriers that asylum seekers and other groups of migrants face [23, 61, 64]. The action-oriented recommendations of this study specify the broader call for cooperation and highlight that, in Germany, especially psychosocial treatment centres and family physicians should be supported to play a more active role in coordination.

Training healthcare providers, particularly family physicians, in cultural competence and in recognizing somatic symptoms as possible manifestations of mental health problems among migrants has been widely emphasized [14, 29, 65–67, 80, 86, 87]. However, as Kietzmann et al. [88] argue, the very notion of cultural competence is contested, and the distinction between general and cultural competence often remains unclear. Conceptualizing culture as a discrete and separate domain within care risks reinforcing stereotypes, rather than promoting genuine intercultural understanding [78, 88]. The suggestions made by the health professionals in this study align with this view, advocating for the integration of anti-discrimination awareness and person-centred approaches into the broader education of all healthcare workers, especially with regard to the needs of marginalized populations. Thus, this study’s findings add an important nuance to the broad demand for cultural competence. Another related recommendation was the promotion of equal opportunities for migrants pursuing careers in healthcare themselves - a proposal supported by previous research, which highlights that increasing the number of health professionals who are migrants themselves enhances intercultural exchange and language diversity in care settings [15, 59, 86].

To address persistent communication barriers, the health professional recommended the institutionalized provision of interpreters and translated materials. This aligns with existing literature, which emphasizes not only the availability of interpreters but also covering their costs through public health insurance [14, 15, 29, 30, 63, 65, 85, 86]. However, reliance on interpreters alone may not always be sufficient [61, 62]. Several studies therefore advocate for the inclusion of cultural mediators, who can bridge both linguistic and cultural gaps [11, 65, 67, 86, 89]. Additional recommendations in the literature include the use of validated assessment tools in multiple languages [14, 62, 86] and implementing new technologies to enhance provider–patient communication [15, 62, 67].

Some authors question the suitability of standard Western therapies for all migrants with mental health problems. They recommend comprehensive care options, such as adapting therapies to individual needs and offering services that also aim to support navigating everyday life in the host country [13, 65, 68, 86], aligning with our study. Another meso-level recommendation of this study in line with the literature is to expand low-threshold options [67, 82]. Geographically accessible mental health services [64, 65, 68], including on-site offers [61] and technology-based interventions [15], are proposed solutions and similar to the ones in our study.

At the micro level, raising mental health awareness is important not just for health professionals and social workers, but also for migrants [15, 67, 68, 82]. Psychoeducation can reduce stigma and improve mental health literacy [14, 61, 68, 90]. In Levesque et al.’s model, (mental) health literacy is considered a determinant of the ability to perceive and can enhance individuals’ awareness of their need for healthcare [46, 57]. This study’s findings propose that such education must be culturally sensitive and include information about the healthcare system and care options [14, 59, 63, 65, 67, 82].

Several studies and guidelines include the promotion of migrants’ mental health as a recommendation for action to improve access [14, 15, 30, 62, 67, 83]. They also mention school-based programs [14, 62, 67]. Many advocate for social support [12, 14, 15, 31, 62, 65, 67], which is another recommendation at micro level. This study’s recommendation to foster social support through intercultural leisure activities appears to be novel in this regard.

### Limitations and strengths

There are some limitations that must be considered when interpreting our study results. The interviews with migrants took place during the COVID-19 pandemic, possibly influencing the findings due to implemented infection control measures. Research suggests that the pandemic has worsened the mental health of various migrant groups [91–93] and increased existing disparities in access to mental healthcare [94, 95], which could limit the generalizability of our findings to post-pandemic contexts. However, the pandemic also provided a unique window into present structural inequalities in access to mental healthcare, as it exacerbated and exposed existing barriers [94, 96, 97]. Additionally, due to triangulation with health professional interviews, the findings are not based solely on data collected during the COVID-19 pandemic.

Furthermore, the interview tool was not explicitly developed to address mental healthcare access, leaving the possibility that not all factors of migrants’ mental healthcare access were discussed. There were also no direct questions asked regarding action-oriented recommendations. However, the interviews were broad in scope and covered many different questions, which allowed these topics to surface. In addition, the secondary analysis was not based on an external dataset. The authors’ context knowledge of the data collection, the interviews, and the characteristics of the participant group of the SonarGlobal project is widely considered an essential advantage for rigorous qualitative secondary analysis [42, 43, 98].

Although the study reflected experiences and viewpoints of three migrant groups whose mental health is under risk because of the intersectionality of different vulnerability mechanisms, their participation was limited due to resource constraints, such as the available language skills of the interviewers. Despite efforts, it was not possible to include more older migrants, as well as more LGBTQ+ persons. Moreover, due to the secondary analysis, data saturation could not be assessed for the interviews with migrants. Future research would also benefit from including additional migrant groups. It is also essential to recognize the heterogeneity within the group of migrants and consider migration-related mental health stressors and individual needs. Instead, the aim was to serve as a starting point for developing comprehensive recommendations for action to improve the overall situation for migrants in Germany.

As a validation meeting with the participating migrants was not feasible, their perspective on the interpretation of their statements is missing. This may have influenced which themes were confirmed or revised, leading to a possible overrepresentation of health professionals’ viewpoints, particularly in the recommendations for action. At the same time, the study followed the advised triangulation with another data source to address the missing participant validation in qualitative secondary analysis, increasing the overall validity of the results [42]: The involvement of health professionals with different specifications and experiences contributed to developing a detailed framework for understanding migrants’ mental healthcare access, which guided the development of recommendations for action. The iterative interviews with health professionals supported the validation of earlier findings and an action-oriented discussion of recommendations, while tailored questions ensured that discipline-specific knowledge gaps were addressed. The findings were also discussed again in several consensus meeting among the research team and with an additional expert. However, the iterative approach may have also directed attention toward topics addressed in previous interviews, potentially influencing the resulting categories. The perspective of experts from disciplines beyond medical professions could be necessary to improve the findings.

## Conclusions

The study’s findings indicate that migrants face numerous obstacles when attempting to access mental healthcare services due to being in a new country, such as difficulties in navigating a new healthcare system, a lack of social support, or the burden of bureaucracy. This is complicated by differences in the perception of mental health and mental disorders. A lack of acceptability and appropriateness of the providers exacerbates the impact of these barriers. However, each of these issues depends on linguistic communication. As a result, language should have special places in an access model for migrants’ mental health. Being not entitled to health insurance for asylum seekers, the lack of available care options and uneven distribution of services further complicates migrants’ access to services.

According to our findings, improving migrant mental healthcare access begins with the removal of all structural barriers caused by discrimination and inequality on macro level. This study highlights that interventions at this level offer opportunities to simultaneously strengthen migrant’s mental health and improve access to healthcare in general. Meso level measures include increasing mental healthcare capacity, improving coordination among system components, and implementing measures to improve service quality, such as increasing provider competencies and providing translation services. This study places particular emphasis on these latter points and offers a new perspective on the role of cultural competency. All of this, however, should be supplemented by measures to promote and raise awareness on mental health for specific groups of migrants on the micro level.

This study is notable for placing a strong focus on migrant perspectives and examining access to healthcare through an intersectional lens, which remains underrepresented in existing research. It reflects that the determinants of mental healthcare access for migrants cannot only be reduced to specific migrant groups. Instead, they are shaped by intersecting dimensions of individual characteristics, structural vulnerabilities, and systemic barriers. Inequalities in healthcare access do not result from a lack of individual capacity, but from structural inequities. This is clearly reflected in this study’s findings for migrants in Munich, Germany, as the recommendations developed in this study offer starting points for interventions at various levels of the healthcare system to address existing structural barriers and improve migrant’s mental healthcare access. The action-oriented recommendations are derived from the determinants identified through the intersectional perspective, providing a context-sensitive basis for potential interventions. The translation into targeted, multi-level recommendations enhances practical relevance and lays the groundwork for further investigation aimed at developing comprehensive recommendations for actions to improve access. With a focus on migrant’s mental healthcare, this study offers updated, action-oriented recommendations in an area where research is still limited in Germany. Mental healthcare access is a fundamental right. This study’s recommendations are an important foundation towards more comprehensive, evidence-based strategies that ensure equitable access to mental health services for all.

## Supplementary Information

Supplementary Material 1. Supplementary Table 1 Recommendations for action to improve migrants' mental healthcare access in five categories.

## Data Availability

Individual privacy prevents the public sharing of interview transcriptions. Codes or categories can be shared on request.
