# Supplementary material for: Towards the development of actionable recommendations for improving mental healthcare access for migrants: a qualitative study in Munich, Germany
Source: BMC Public Health. 2026 Feb 25;26:1074. doi: 10.1186/s12889-026-26673-1 (PMC13040945; doi:10.1186/s12889-026-26673-1)
Supplement: Supplementary file 1 — Supplementary Material 1. Supplementary Table 1 Recommendations for action to improve migrants' mental healthcare access in five categories. [file 12889_2026_26673_MOESM1_ESM.docx]

**Supplementary Table 1** Recommendations for action to improve migrants' mental healthcare access in five categories

| **Category 1. Enhance the structure, organization, and funding of (mental) healthcare** |
| --- |
| 1. Enhance coordination between healthcare and public institutions through a central contact point for providers and migrants |
| 1. Increase funding, capacity, and distribution of psychosocial treatment centres |
| 1. Incorporate more low-threshold options, such as self-help groups, digital health applications, and on-site services into the healthcare system |
| 1. Expand comprehensive care options specific for migrants, such as family therapies, language development, body awareness and occupational therapies |
| 1. Reduce the language barrier by using translations and providing interpreters, including their cost coverage by health insurance. |
| **Category 2.** **Empower healthcare providers** |
| 1. Empower family physicians by raising awareness, implementing referral programs with health insurances, and offering specialized training in mental health treatment. |
| 1. Integrate the topic of migrant (mental) health into the curriculum of all medical professions |
| 1. Facilitate access to medical professions for migrants by ensuring equal opportunities in training and targeted encouragement |
| **Category 3.** **Facilitate the adaptation to a new healthcare system** |
| 1. Provide education about the German healthcare system to migrants early after arrival |
| 1. Disseminate information on mental healthcare providers and treatment options through analogue and digital channels |
| 1. Educate migrants for sensitization and self-management of mental health problems, as well as the reasons for seeking external help |
| **Category 4. Enhance social and organizational support** |
| 1. Raise awareness and allocate resources on organizations providing support for migrants |
| 1. Promote social support networks to combat social isolation among migrants |
| 1. Offer intercultural activities that foster social support and challenge prejudices |
| **Category 5.** **Take into account determinants of health** |
| 1. Promote the mental well-being and integration of children and adolescents from migrant families by offering leisure activities besides academic achievement in schools |
| 1. Eliminate living conditions for asylum seekers that violate human rights, including enhanced privacy, access to leisure activities, WiFi connection, as well as the acceleration of the decision-making process on their residence status to reduce psychological stress and ensure continuous mental healthcare |
| 1. Ensure equal treatment for all migrants regardless of their origin |
